# Molecular Basis of Aggressiveness in Pituitary Adenomas and Its Association With the Immune Microenvironment

**DOI:** 10.1155/ijog/9346050

**Published:** 2025-07-17

**Authors:** Xiaoyan Chen, Jingnan Wang, Qianqian Guo

**Affiliations:** ^1^National Cancer Center/National Clinical Research Center for Cancer/Cancer Hospital, Chinese Academy of Medical Sciences and Peking Union Medical College, Beijing, China; ^2^Department of Radiation Oncology, The First Affiliated Hospital of Bengbu Medical College, Bengbu, China; ^3^Department of Oncology, The First Affiliated Hospital of Zhengzhou University, Zhengzhou, China

**Keywords:** diagnostic marker, differential expression analysis, immune infiltration, pituitary adenomas, receiver operating characteristic curve, weighted gene coexpression network analysis

## Abstract

**Background:** Pituitary adenomas (PAs) are common intracranial tumors, and their aggressive phenotype exhibits a poor prognosis. We aimed to explore the aggressive feature of PAs and discover novel diagnostic markers.

**Method:** The datasets of GSE260487 and GSE169498, which contained invasive and noninvasive samples, were downloaded from the Gene Expression Omnibus (GEO) database. Aggressive phenotype-related gene modules were classified using the “WGCNA” package. Differentially expressed genes (DEGs) in each module were identified by the “limma” package. Next, a protein–protein interaction (PPI) network was used in the construction and identification process of key genes, and the CytoHubba tool was utilized to analyze the subnetwork and select the top 10 genes. Diagnostic markers were selected using two machine learning algorithms: support vector machine (SVM) and Lasso. Finally, the ESTIMATE and “GSVA” were applied for immune infiltration assessment.

**Results:** WGCNA showed that the turquoise module was closely associated with the aggressive phenotype and enriched in neural differentiation and cell migration pathways. A total of 521 DEGs were intersected with the turquoise module genes to obtain 187 overlapping genes, from which 10 hub genes related to tumor proliferation were selected to develop a PPI network. Next, we determined *MYH7* as an accurate diagnostic marker, and the immune infiltration analysis revealed that *MYH7* expression was negatively correlated with stromal score and immune score but positively correlated with the infiltration of antitumor cells.

**Conclusion:** We developed a novel marker with a strong diagnostic performance for PAs, providing novel insights for the detection and individualized treatment of PAs.

## 1. Introduction

Human pituitary adenoma (PA) is the third most prevalent intracranial neoplasms following meningiomas and gliomas, accounting for 17% of all brain tumors [1, 2]. While the majority of PA cases are benign, PA frequently causes clinically significant symptoms due to the overproduction of pituitary hormones and/or local occupation of the tumor mass [3]. Based on the clinical manifestations, PA could be classified into the following subtypes: prolactinomas (PRL, also known as prolactin-secreting PAs), clinically nonfunctioning pituitary adenomas (NFPAs), growth hormone (GH)–secreting adenomas, adrenocorticotropin hormone (ACTH)–secreting adenomas (also known as Cushing's disease), and thyroid-stimulating hormone (TSH)–secreting adenomas. Some other rare PAs produce clinically relevant luteinizing hormone (LH) and follicle stimulating hormone (FSH) [3]. Current therapies for PAs are limited, with somatostatin analogues and dopamine agonists as two major drugs for the treatment of PAs. Specifically, dopamine agonists are usually the first choice for hormonal and tumor size control in PRL-secreting PAs, while somatostatin analogues are often used for GH-producing PAs and post-transsphenoidal surgery treatment [3]. However, not all PA patients can benefit from these methods, which demands the identification of potential effective therapeutic biomarkers for individualized treatments.

PAs can be divided according to size as microadenomas (< 10 mm), macroadenomas (≥ 10 mm), or giant adenomas (≥ 30 mm). Based on invasiveness, PAs can be further divided into noninvasive PAs and invasive PAs. A study revealed that 25%–55% of PAs exhibit invasion to bone, dura, or surrounding anatomical structures [4]. However, most noninvasive PAs have benign behaviors even with dural invasion. A study reported that the incidence of malignant pituitary tumors with systemic and meningeal metastases is notably low (0.2%) [5]. In addition, some invasive PAs with highly aggressive characteristics display distinct clinical behaviors in comparison to benign PAs and and are characterized by the invasion of nearby anatomical structures, early postoperative recurrence, and treatment resistance. The World Health Organization (WHO) has published a classification criterion that, in addition to benign and malignant PAs, invasive PA, a type of atypical adenoma, exhibits aggressive behaviors, high mitotic index, extensive p53 nuclear staining, and Ki67 labeling index (LI) > 3% [6]. It has been found that invasive PA shows carcinoma morphological and histological features and has craniospinal or distant metastases, fatal outcomes, and poorer prognosis. Therefore, the investigation of the underlying pathogenesis of PAs with aggressive characteristics could facilitate the discovery of novel molecular targets and improvement of the prognosis of PAs [7].

This study downloaded both invasive and noninvasive PA samples from public databases and conducted weighted gene coexpression network analysis (WGCNA) to explore the aggressive features of PAs. The “limma” R package was used for filtering differentially expressed genes (DEGs), which were then intersected with aggressive genes in the module. The common genes were subjected to support vector machine (SVM) and Lasso analyses to determine potential diagnostic markers. Finally, we used the receiver operating characteristic (ROC) curve to assess the diagnostic efficiency of the identified biomarkers, and the correlation between the biomarkers and immune infiltration was also investigated. Our findings provided some insights for the diagnosis and individualized treatment of aggressive PAs.

## 2. Materials and Methods

### 2.1. Data Sources

Datasets of GSE260487 and GSE169498 were obtained from the Gene Expression Omnibus (GEO) database (https:/http://www.ncbinlm.nih.gov/geo/) [8]. Specifically, GSE260487 included 15 invasive PA samples and 17 noninvasive PA samples, and GSE169498 contained 49 invasive and 24 noninvasive PA samples.

### 2.2. Differential Expression Analysis

For identifying the DEGs, we applied the “limma” R package to perform differential expression analysis between invasive and noninvasive PA samples in the GSE169498 cohort [9], with adj.*p* val < 0.05 and |log_2_FC| > 1 as the threshold value [10].

### 2.3. WGCNA

WGCNA was used to identify gene modules linked to aggressive phenotype in the GSE169498 cohort using the “WGCNA” R package [9]. Firstly, the optimal soft threshold (*β*) was determined by the pickSoftThreshold function for scale independence and adjacent mean analysis to ensure a scale-free network (log(k) was positively correlated with the log(P(k)) and the correlation coefficient was greater than 0.85). Then, the expression matrix was converted into an adjacency matrix, which was further transformed to a topological matrix (TOM). Hierarchical clustering (average-linkage) was performed for identifying coexpression modules based on the optimal soft threshold (*β*) and TOM. After using the dynamic shear method to identify gene modules, the eigengenes of each module were calculated for further clustering analysis, and the modules with close distance were merged into a new module under height = 0.25, deepSplit = 2, and min ModuleSize = 50 [11].

### 2.4. Protein–Protein Interaction (PPI) Network Analysis

STRING (https://cn.string-db.org/, Version 12.0), a PPI network retrieval tool, was used to analyze the genes. Corresponding PPI networks were visualized using Cytoscape software (Version 3.9.1), and the CytoHubba algorithm was employed for identifying the subnet [12, 13].

### 2.5. Machine Learning for Screening Diagnostic Markers

The recursive feature elimination (RFE) function with svmLinear method in the “caret” R package and the 10-fold CrossValidation (cv) algorithm was applied to screen the key markers [14]. Lasso regression of “glmnet” R package was used for identifying the features (key parameters: nfolds = 10, family = “binomial”) [14]. Then, the diagnostic performance of the markers was assessed based on the ROC curve using the “timeROC” R package [15].

### 2.6. Immune Infiltration Analysis

Immune infiltration was evaluated using the ESTIMATE method [7]. Single-sample gene set enrichment analysis (ssGSEA) scores of 28 types of tumor-infiltrating lymphocytes (TILs) were calculated based on the marker genes from a previous study utilizing the “GSVA” R package [16].

### 2.7. Statistical Analysis

All statistical data were analyzed in R language (Version3.6.0). Wilcoxon's test was used to calculate the difference between two sets of continuous variables. The correlation matrix was developed by the Pearson method. The data with *p* < 0.05 were considered statistically significant (⁣^∗^*p* < 0.05, ⁣^∗∗^*p* < 0.01, ⁣^∗∗∗^*p* < 0.001, and ⁣^∗∗∗∗^*p* < 0.0001).

## 3. Results

### 3.1. Identifying the Aggressive Feature-Related Gene Modules for the PA Samples

**Figure 1:**
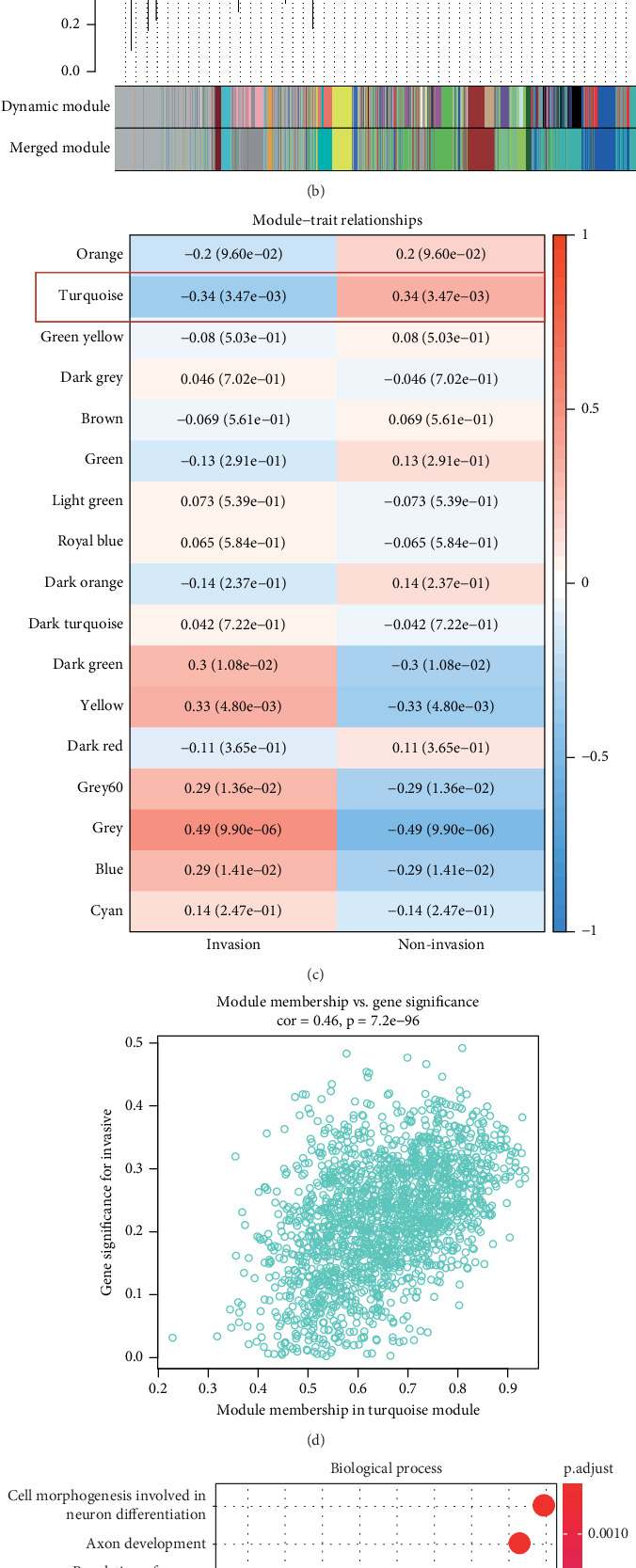
WGCNA of invasiveness-related gene module. (a) Scale independence and the adjacent mean plot for the optimal soft threshold. (b) Hierarchical clustering for the gene module. (c) Heatmap of correlations between different gene modules and invasive and noninvasive traits. (d) Correlation analysis between gene member values and gene significance in the turquoise module. (e) Biological process enrichment analysis of the genes in the turquoise module.

WGCNA was performed to identify gene modules linked to the invasiveness phenotype. The scale independence and adjacent mean plot showed that the scale-free topological fitting index was stable when the soft threshold was 12 (Figure 1). Based on the soft threshold, hierarchical clustering of the genes was performed for classifying the coexpression gene modules (Figure 1). Correlation analysis demonstrated that the turquoise module was significantly closely associated with the invasiveness phenotype (*p* < 0.05, Figure 1). Enrichment analysis in biological process (BP) term of the turquoise module genes demonstrated that the module was significantly enriched in several BPs related to neural differentiation and cell migration, including cell morphogenesis involved in neuron differentiation, axon development, and organic anion transport process (Figure 1).

### 3.2. Screening Key Genes

**Figure 2:**
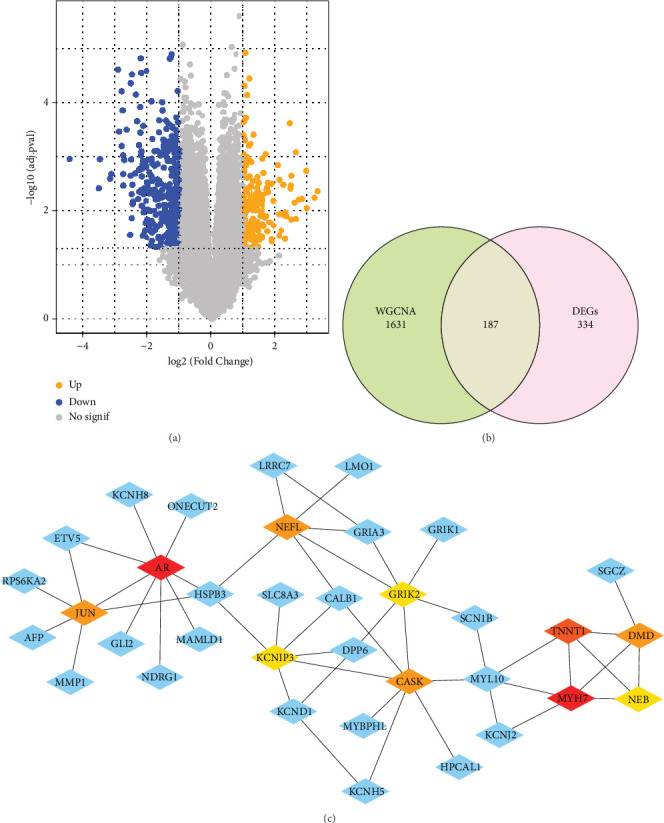
Differential expression analysis and protein–protein interaction (PPI) network. (a) Volcano plot of the DEGs. (b) Venn plot of the overlapping genes between the DEGs and WGCNA. (c) The PPI network for the key genes (red and orange represent the core genes with the highest scores).

A sum of 521 DEGs in the GSE169498 cohort was identified (Figure 2a). There were 187 overlapping genes in the intersection between the turquoise module genes and the DEGs (Figure 2b). Subsequently, based on the PPI network, we identified the Top 10 key genes from the 187 overlapping genes (Figure 2c), including *AR*, *JUN*, *NEFL*, *MYH7*, *KCNIP3*, *GRIK2*, *CASK*, *TNNT1*, *NEB*, and *DMD*. These genes may play an important role in the aggressive progression of PAs.

### 3.3. Machine Learning Algorithms Selected Potential Diagnostic Markers

**Figure 3:**
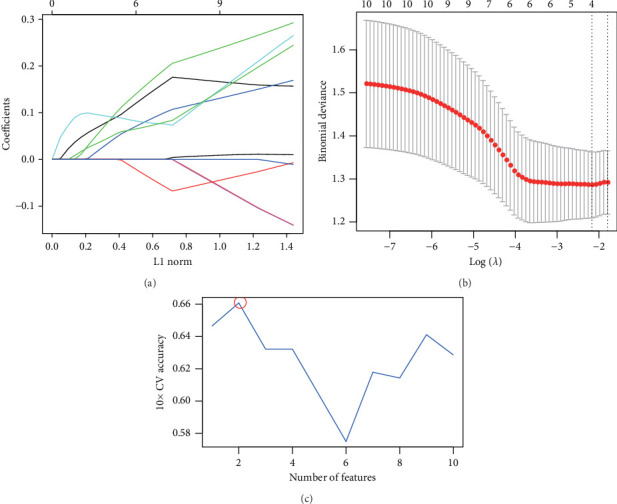
Machine learning screening for the key genes. (a) Lasso coefficient path and regularization path plot for the number of candidate genes. (b) Support vector machine (SVM) algorithm for the number of candidate genes. (c) Venn plot of the overlapping genes from the Lasso and SVM models.

The aforementioned 10 key genes were used for further analysis. Lasso Cox regression analysis was used for selecting the key candidate genes. The *λ* for the optimal number of feature genes in the model was determined according to the Lasso coefficient and regularization trajectories (Figure 3). Further, the RFE method was used to select the number of features, and the SVM model determined that the model had the highest cross-verification accuracy when the number of features was 4 (Figure 3). Finally, we identified *MYH7* as the diagnostic marker based on the results of the two machine learning algorithms (Figure 3).

### 3.4. MYH7 Was the Biomarker With a High Diagnostic Efficiency

**Figure 4:**
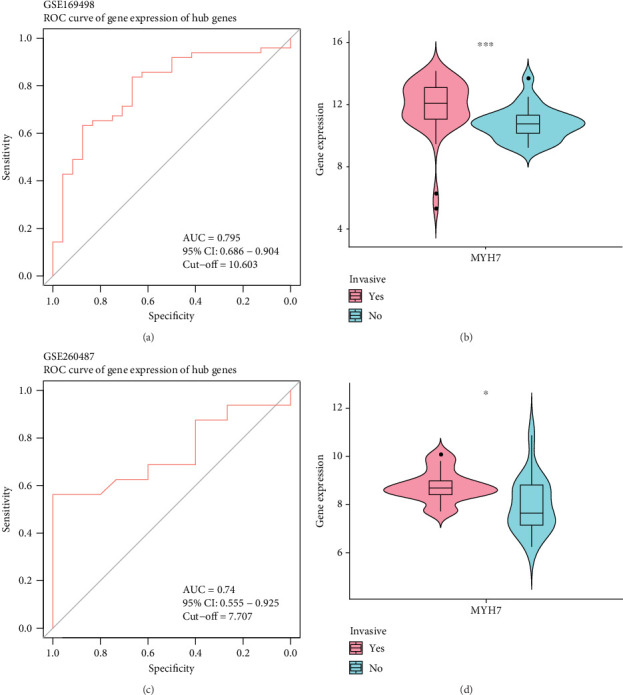
Establishment and verification of diagnostic model. (a) Receiver operating characteristic curve for the diagnostic accuracy in the GSE169498 cohort. (b) The expression difference of the diagnostic gene in invasive and noninvasive groups in the GSE169498 cohort. (c) Receiver operating characteristic curve for the diagnostic accuracy in the GSE260487 cohort. (d) The expression difference of the diagnostic gene in the GSE260487 cohort (⁣^∗^*p* < 0.05 and ⁣^∗∗∗^*p* < 0.001).

The diagnostic efficiency of *MYH7* in the GSE169498 cohort was analyzed through the ROC analysis. The results showed that the area under curve (AUC) value of the diagnostic marker was 0.8 (Figure 4a), indicating a high classification effectiveness of *MYH7* in PA diagnosis. Meanwhile, we observed that the expression level of *MYH7* was significantly upregulated in the invasive phenotype group (*p* < 0.05, Figure 4b). Consistent results were observed in the validation set of GSE260487, with an AUC = 0.74 and significantly overexpressed *MYH7* in the invasive group (*p* < 0.05, Figure 4c,d).

### 3.5. MYH7 May Promote the Invasive Properties of PAs by Influencing the Function of Antitumor Cells

**Figure 5:**
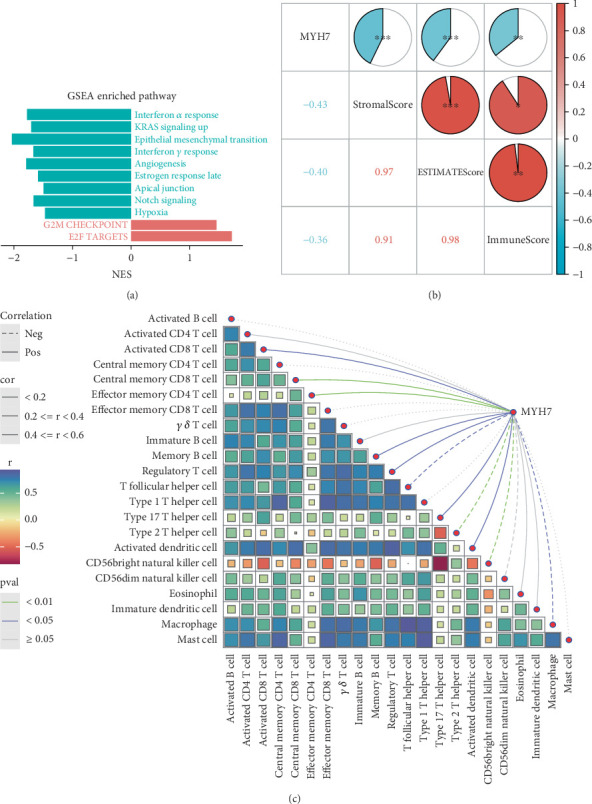
Potential relationship between PA-related diagnostic markers and the tumor immune microenvironment. (a) GSEA results for invasive versus noninvasive PA samples. (b) Correlation analysis between *MYH7* expression levels and tumor microenvironment scores. (c) Correlation between *MYH7* gene expression and ssGSEA immune score in invasive pituitary tumors. (⁣^∗^*p* < 0.05, ⁣^∗∗^*p* < 0.01, and ⁣^∗∗∗^*p* < 0.001).

Gene set enrichment analysis (GSEA) of invasive and noninvasive pituitary tumors revealed that proliferation-related pathways like G2M CHECKPOINT and E2F TARGETS were significantly enriched in the invasive samples (Figure 5). After calculating the immune infiltration score, correlation analysis revealed that the expression of *MYH7* was significantly negatively correlated with the StromalScore, ImmuneScore, and ESTIMATEScore, with a correlation coefficient of −0.43, −0.36, and −0.40, respectively (*p* < 0.01, Figure 5), indicating that the expression of *MYH7* was associated with the suppressive tumor microenvironment of the invasive PAs. Additionally, the correlation analysis between the expression of *MYH7* and the infiltration of a variety of immune cells demonstrated that *MYH7* was positively correlated with CD8 T cells, memory B cells, and regulatory T cells but was negatively correlated with T helper cell, Type 2 T helper cell, and macrophage in the invasive PA samples (*p* < 0.05, Figure 5). This suggested that *MYH7* may promote the invasiveness of PAs by influencing the function of antitumor cells.

## 4. Discussion

PAs are common intracranial neoplasms originating from adenohypophysis cells. Somatostatin analogues and dopamine agonists are the first- or second-line drugs for PAs [17]. Furthermore, the clinical treatment of many PAs remains difficult due to the invasive nature of the tumors. In some cases, the tumor is unresectable or is too small to be detected. Moreover, stereotactic radiosurgery with adjuvant chemotherapy is not completely effective in achieving biochemical remission and tumor control. A study reported that the 10-year recurrence rate of the tumor is 7%–12% [18]. Therefore, this study explored the aggressive features of PAs to discover potential biomarkers for its diagnosis and treatment [19].

After screening the DEGs and performing PPI analysis, *AR*, *JUN*, *NEFL*, *MYH7*, *KCNIP3*, *GRIK2*, *CASK*, *TNNT1*, *NEB*, and *DMD* were selected as the hub genes in the aggressive PA samples. The high expression of *AR* mediated the PRL-PA dopamine agonist resistance through reducing the level of reactive oxygen species (ROS) [20]. *JUN* is a proto-oncogene that regulates cell proliferation and apoptosis [21], and *NEFL* could promote the invasion and migration of esophageal squamous carcinoma through activating the EGFR/AKT/S6 pathway [22]. Silencing of *KCNIP3* enhances the epithelial–mesenchymal transition and proliferation via activating the Wnt/*β*-catenin pathway in papillary thyroid carcinoma [23]. *GRIK2* maintains the function of urothelial carcinoma stem-like cells [24]. *CASK* acts as a tumor promoter in pancreatic cancer [25]. *TNNT1* is regulated by miR-873 to promote colorectal cancer progression [26]. These findings suggested that the screened hub genes were closely associated with the migration and invasion of PAs and could serve as potential target genes for the treatment of PAs.

*MYH7* encodes the heavy chain of myosin and plays an important role in cardiac and skeletal muscle contraction [27]. Variation of *MYH7* can cause hypertrophic cardiomyopathy (HCM) [28], and *MYH7* has also been considered the hub gene in oral cancer [29]. We utilized two machine learning algorithms to identify *MYH7* as a diagnostic marker with a high diagnostic accuracy in PAs. Similar findings in a previous study revealed that downregulated *MYH7* is a biomarker for the poor prognosis of head and neck squamous cell carcinoma (HNSCC), and that *MYH7* promotes CD4+ T cell activation in cancer [30]. In addition, we also found that the expression of *MYH7* was negatively correlated with the stromal score and immune score and positively correlated with the infiltration of antitumor cells, including CD8 T cells and Memory B cells. This indicated that the expression of *MYH7* aggravated the exhaustion of antitumor immune cells for a tumor-suppressive microenvironment, which may contribute to the aggressive phenotype of PAs. Overall, we identified *MYH7* as a novel diagnostic marker with a high diagnostic accuracy for PAs, and *MYH7* may be associated with a tumor-suppressive microenvironment through promoting the exhaustion of immune cells.

## 5. Conclusion

The present study explored the aggressive features of PA patients and constructed a PPI network containing the key hub genes with the potential to serve as the therapeutic targets for PAs. Importantly, *MYH7* was identified as an accurate diagnostic marker for PAs, providing some novel insight for further study.

### 5.1. Limitation

There were some limitations in this study. First, the study only used two public datasets (GSE169498 and GSE260487) with a small sample size and limited data sources, which may influence the robustness of *MYH7* as a diagnostic factor. In the future, we will validate the expression of *MYH7* and its diagnostic efficacy by including independent multicenter clinical samples and large-scale patient clinical follow-up data and by measuring its protein level. In addition, this study identified *MYH7* as a key gene related to the aggressive phenotype of PAs employing two machine learning algorithms, but we lacked in vitro or in vivo experiments to verify its specific function in PA cells. Follow-up studies will use cell line and animal models to overexpress and interfere with *MYH7* as well as cell function experiments to further clarify its mechanism of action in PAs. Finally, this study only used the ssGSEA algorithm to analyze the immune cell infiltration characteristics, but this method does not provide data about the spatial distribution in real tumor tissues and cannot distinguish the functional status of immune cells. Therefore, further studies will employ spatial transcriptomics or single-cell RNA sequencing techniques to analyze the spatial relationship between *MYH*7 expression region and the location of immune cell infiltration and their association with immune escape or depletion status.

## Data Availability

The datasets generated and/or analyzed during the current study are available in the GSE260487 repository (https://www.ncbi.nlm.nih.gov/geo/query/acc.cgi?acc=GSE260487) and the GSE169498 repository (https://www.ncbi.nlm.nih.gov/geo/query/acc.cgi?acc=GSE169498).

## Nomenclature

PAs: pituitary adenomas
DEGs: differentially expressed genes
PPI: protein–protein interaction
PRL: prolactin
WHO: World Health Organization
WGCNA: weighted gene coexpression network analysis
SVM: support vector machine
Lasso: least absolute shrinkage and selection operator
ROC: receiver operating characteristic
GEO: Gene Expression Omnibus database
ssGSEA: single sample gene set enrichment analysis
